# Determining the changes in metabolites of *Dendrobium officinale* juice fermented with starter cultures containing *Saccharomycopsis fibuligera* FBKL2.8DCJS1 and *Lactobacillus paracasei* FBKL1.3028 through untargeted metabolomics

**DOI:** 10.1186/s12866-023-02807-y

**Published:** 2023-03-14

**Authors:** Wanlin Liu, Xiaoye Luo, Shuyi Qiu, Wu Huang, Yanan Su, Linling LI

**Affiliations:** 1 College of Liquor and Food Engineering, Guizhou University, Guiyang, 550025 China; 2 Guizhou Provincial Key Laboratory of Fermentation and Biophomacy, Guizhou University, Guiyang, 550025 China; 3 College of Life Sciences, Guizhou University, Guiyang, 550025 China

**Keywords:** *D.officinale* juice, Volatile components, Differential metabolites, Untargeted metabolomics

## Abstract

**Background:**

The present study aimed to investigate the changes in volatile components and metabolites of *Dendrobium officinale* (*D. officinale*) juice fermented with starter cultures containing *Saccharomycopsis fibuligera* and *Lactobacillus paracasei* at 28 ℃ for 15 days and post-ripened at 4 ℃ for 30 days using untargeted metabolomics of liquid chromatography-mass spectrometry (LC–MS) and headspace solid-phase microextraction-gas chromatography (HS–SPME–GC–MS) before and after fermentation.

**Results:**

The results showed that the alcohol contents in the *S. fibuligera* group before fermentation and after fermentation were 444.806 ± 10.310 μg/mL and 510.999 ± 38.431 μg/mL, respectively. Furthermore, the alcohol content in the fermentation broth group inoculated with the co-culture of *L. paracasei* + *S. fibuligera* was 504.758 ± 77.914 μg/mL, containing a significant amount of 3-Methyl-1-butanol, Linalool, Phenylethyl alcohol, and 2-Methyl-1-propanol. Moreover, the Ethyl L (-)-lactate content was higher in the co-culture of *L. paracasei* + *S. fibuligera* group (7.718 ± 6.668 μg/mL) than in the *L. paracasei* (2.798 ± 0.443 μg/mL) and *S. fibuligera* monoculture groups (0 μg/mL). The co-culture of *L. paracasei* + *S. fibuligera* significantly promoted the metabolic production of ethyl L (-)-lactate in *D. officinale* juice. The differential metabolites screened after fermentation mainly included alcohols, organic acids, amino acids, nucleic acids, and their derivatives. Twenty-three metabolites, including 11 types of acids, were significantly up-regulated in the ten key metabolic pathways of the co-culture group. Furthermore, the metabolic pathways, such as pentose and glucuronate interconversions, the biosynthesis of alkaloids derived from terpenoid and polyketide, and aminobenzoate degradation were significantly up-regulated in the co-culture group. These three metabolic pathways facilitate the synthesis of bioactive substances, such as terpenoids, polyketides, and phenols, and enrich the flavor composition of *D. officinale* juice.

**Conclusions:**

These results demonstrate that the co-culture of *L. paracasei* + *S. fibuligera* can promote the flavor harmonization of fermented products. Therefore, this study provides a theoretical basis for analyzing the flavor of *D. officinale* juice and the functional investigation of fermentation metabolites.

## Background

Fermentation is a metabolic process characterized by the release of different chemicals (metabolites) through the action of microorganisms. Lactobacillus and yeast, widely known probiotics, are the most common microorganisms involved in food fermentation [1]. For instance, *Lactobacillus Rhamnosus, Lactobacillus Plantarum,* and yeast co-cultures can inhibit the growth of pathogenic bacteria, neutralize enterotoxins, and develop antibiotic resistance [2, 3]. The co-culture of lactic acid bacteria and yeasts can enhance sugar metabolism and improve aroma formation during the fermentation of cocoa beans [4]. Yeast provides vitamins, amino acids, and other necessary growth factors for lactic acid bacteria during fermentation. The end products of lactic acid bacteria metabolism are utilized as an energy source by the yeasts [5]. The synergistic effect of these microorganisms in the fermentation process can improve the palatability, sensory properties, utilization rate of nutrients in food and enhance food safety [6].

Chinese medicine has been based on the theory that "medicine and food have the same origin, " meaning that many foods can act as medicines with equivalent effectiveness in preventing and treating diseases. Many medicinal plants, such as *Radix Codonopsis, D. officinale, Astragalus, Ganoderma, Amanita, Cornu Cerevisiae,* and *Eucommiae*, have been used in the health food industry, In 2020, China listed Dendrobium officinale as a medicinal and edible plant. [7]. As a traditional Chinese medicine widely used in many Asian countries, D. officinale is also considered a promising source of medicinal plants for the discovery of novel natural products with significant chemical and biological properties [8].,*D. officinale* is one of the common medicinal food plants containing various bioactive ingredients, such as polysaccharides, bibenzoids, phenolics, alkaloids, and flavonoids. Research has shown that *D. officinale* possesses anti-tumor, gastrointestinal protection, hypoglycemic, anti-aging, and anti- osteoporosis effects [9]. Tian et al. fermented *D. officinale* with Bacillus sp. DU-106 and found that DU-106 can change the monosaccharide composition and molecular weight of *D. officinale* polysaccharides and promote immune stimulating activity [10]. However, the current research on *D. officinale* is mainly focused on the extraction and application of natural bioactive components, with only a few reports on the fermentation of *D. officinale* using probiotics [11].

Metabolite changes during microbial fermentation have been recently studied using untargeted metabolomics methods, which can characterize many low molecular weight and low concentration compounds and infer the functions and metabolic pathways of metabolites. Untargeted metabolomics methods can also screen and analyze the differential metabolites before and after fermentation. Therefore, untargeted metabolomics methods can be used to analyze metabolite changes during liquid fermentation of *D. officinale* with Yeast and Lactobacillus[12, 13].

In this study, *D. officinale*, as a raw material, was inoculated with lactic acid bacteria and yeast for liquid fermentation. The changes and differences in compounds before and after fermentation of *D. officinale* juice were investigated using headspace solid-phase microextraction-gas chromatography–mass spectrometry and untargeted metabolomics liquid chromatography-mass spectrometry combined with multivariate statistical analyses. Therefore, this study may provide insights into analyzing the functional and aroma characteristics of *D. officinale* juice.

## Results

### HS–SPME–GC–MS analysis

**Fig. 1:**
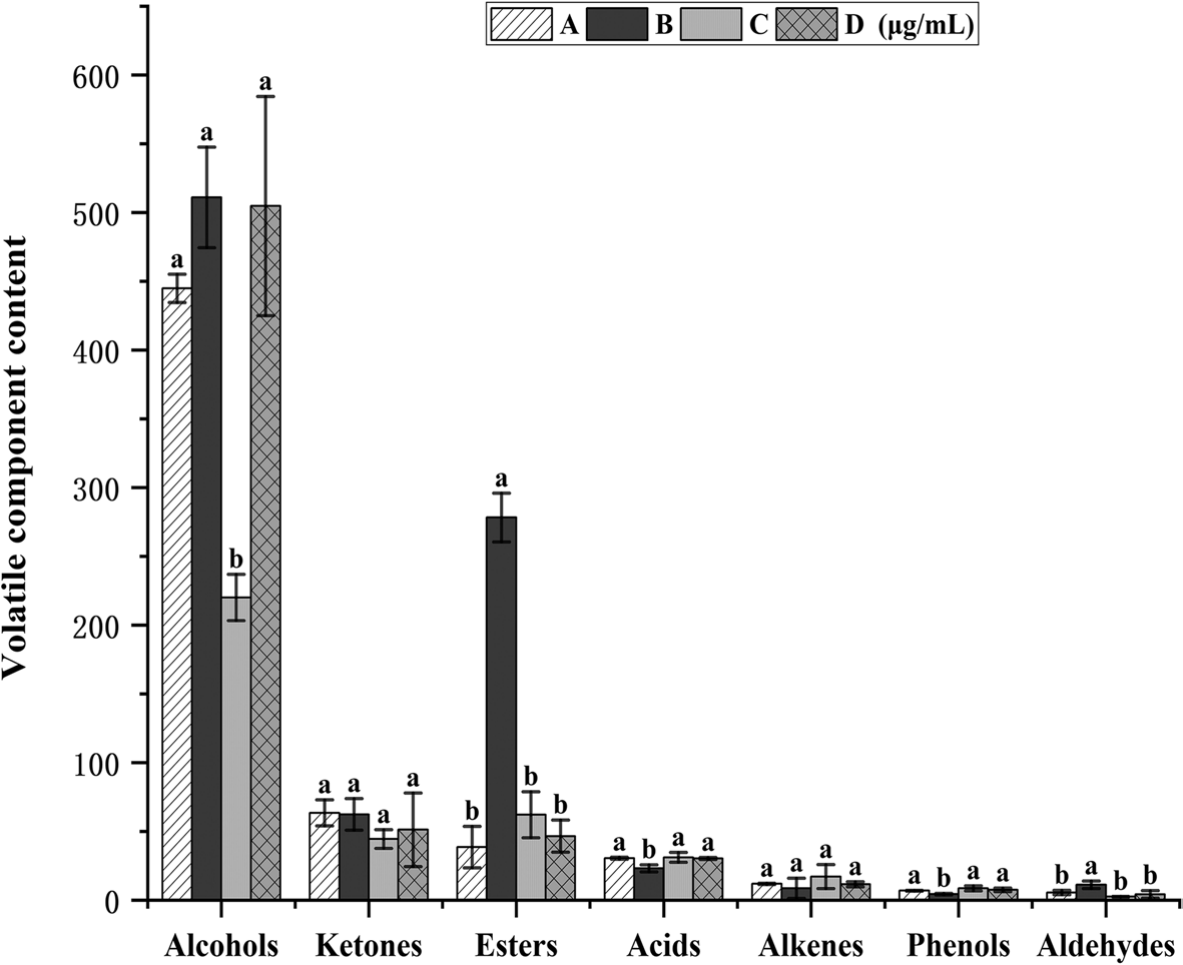
Relative volatile components of D. officinale juice before and after fermentation. (The unfermented D. officinale juice (**A**), S. fibuligera fermented D. officinale juice (**B**), L. paracasei fermented D. officinale juice (**C**), L. paracasei + S. fibuligera fermented D. officinale juice (**D**))

HS–SPME–GC–MS detected 50, 40, 50, and 49 volatile component species of *D. officinale* juice (alcohols, esters, ketones, acids, phenols, and aldehydes) in the unfermented group, single *S. fibuligera* group, single *L. paracasei* group, and *S. fibuligera* + *L. paracasei* group, respectively (Fig. 1). The alcohol content in the stock solution was 444.806 ± 10.310 μg/mL, which significantly decreased to 220.093 ± 21.340 μg/mL after single *L. paracasei* fermentation. The total content of volatile components was reduced by 216.546 ± 35.725 μg/mL after single *L. paracasei* fermentation. *S. fibuligera* fermentation significantly increased the alcohol content to 510.999 ± 38.431 μg/mL and the contents of linalool, phenyl ethanol, isobutanol, and alpha-pinoresinol. The primary lipid produced after *S. fibuligera* fermentation was isoamyl formate (236.473 ± 12.868 μg/mL). The alcohol content after *D. officinale* juice inoculation with *L. paracasei* + *S. fibuligera* increased to 504.758 ± 77.914 μg/mL, with isoamyl alcohol nearly twice as much as the original solution. This inoculation enhanced the taste of *D. officinale* juice and positively improved the aroma complexity [14]. These results indicate that single *S. fibuligera* fermentation and simultaneous inoculation of *L. paracasei* + *S. fibuligera* can increase the alcohol content. However, *when fermented alone, S. fibuligera did not produce ethyl L(-)-lactat*e. Compared with *L. paracasei* fermentation, the amount of ethyl L(-)-lactate significantly increased after mixed culture fermentation (three times higher), indicating that the co-culture of these two strains promotes the metabolic production of ethyl L (-)-lactate. The primary esters in the unfermented *D. officinale* juice included ethyl caproate and isoamyl acetate, while the primary esters in the fermented samples were ethyl cinnamate, ethyl caproate, ethyl octanoate, isoamyl formate, and ethyl L(-)-lactate. This result indicates that the short-chain esters were gradually converted to long-chain esters via microbial metabolism of *D. officinale* juice [15]. Furthermore, fermentation with inoculated strains altered volatile compound distribution (Fig. 1) [16]. Acids play a vital role in fermented beverages by acting as precursors in the synthesis of compounds, such as esters, alcohols, and methyl ketones. Esters are produced through the esterification of organic acids with alcohols or the metabolism of amino acids [17]. As we all know, HS-SPME-GCMS was used to detect volatile compounds.However the total acid compounds produced by *L. paracasei* and *S. fibuligera* of fermentation metabolism were mainly organic acids,And their main detection method that the researchers used were HPLC and LC–MS.

**Fig. 2:**
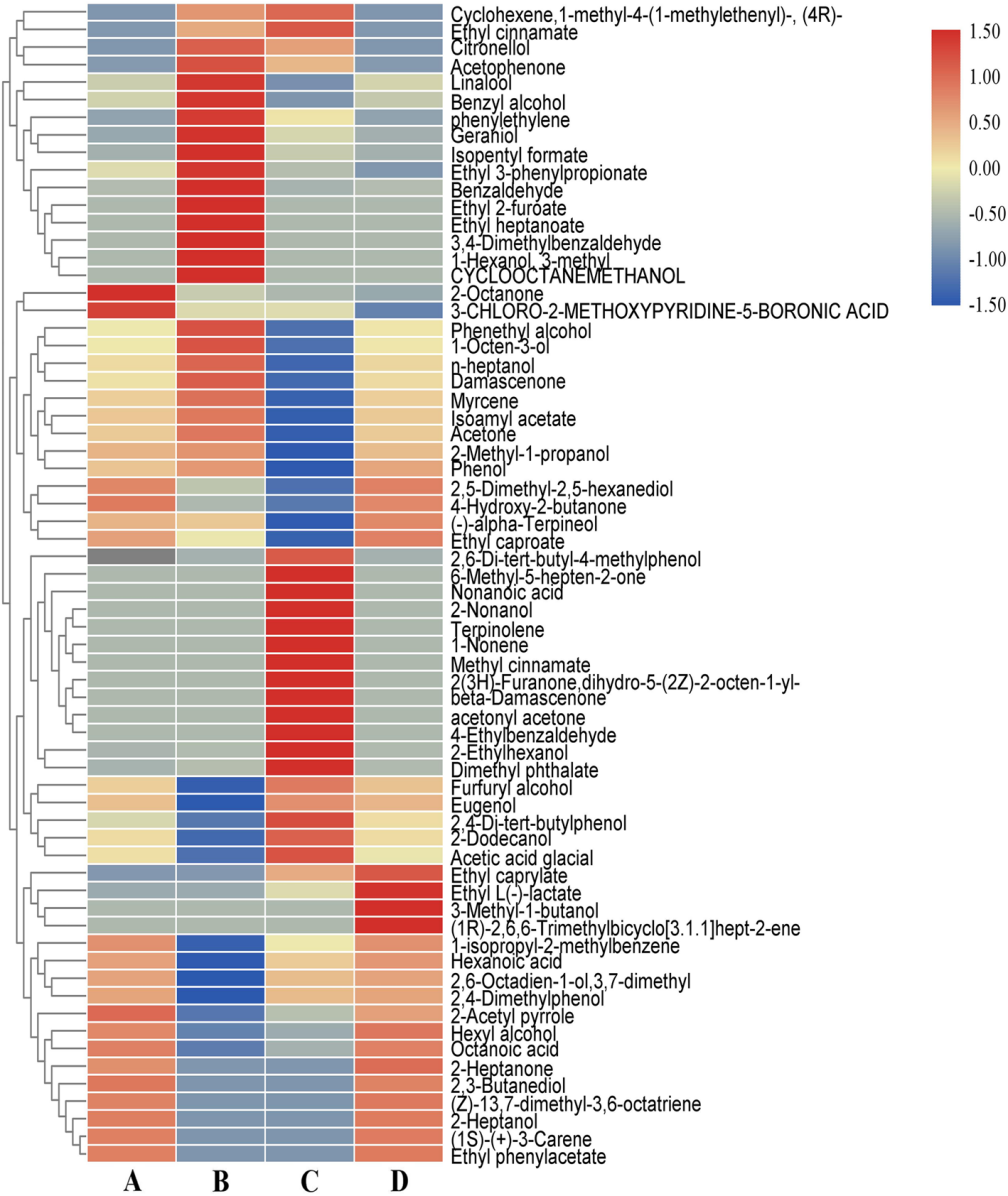
Heat map of the volatile components in D. officinale juice with different inoculations

The changes in specific volatile compounds before and after fermentation are shown in the thermogram in Fig. 2. The volatile components in group A (unfermented *D. officinale* juice) mainly included 2-octan, 3-chloro-2-methoxypyridine-5-boronic acid, 2,5-dimethyl-2,5-hexanediol, 4-hydroxy-2- butanone, and 1-isopropyl-2-methylbenzene. The volatile components in group B (*D. officinale* juice fermented with *S. fibuligera*) mainly included citronellol, acetophenone, linalool, benzyl alcohol, phenylethylene, geraniol, isoamyl acetate, ethyl 3-phenylpropionate, benzaldehyde, ethyl 2-furoate, ethyl heptanoate, 3,4-dimethyl benzaldehyde, 3-methyl-1-hexanol, cyclooctene, phenyl ethanol, 1-octen-3-ol, heptanol, and dammarone. The volatile components in group C of *D. officinale* juice fermented by *L.paracasei* mainly included 2,6-Di-tert-butyl-4- methyl phenol, methylheptenone, nonanoic acid, 2-nonanol, terpinolene, 1-nonene, methyl cinnamate, 2(3H)-Furanone, dihydro-5-(2Z)-2-octen-1-yl-, beta-damascenone, acetyl acetone, 4-ethylbenzaldehyde, 2-ethyl hexanol, dimethyl phthalate, 2,4-di-tert butyl phenol, 2-dodecanol, and acetic acid. The volatile components in group C of *D. officinale* juice fermented with the mixed culture of *L. paracasei* and *S. fibuligeraei* mainly included ethyl n-hexanoate, ethyl L (-)-lactate, isopentane, and 6,6-trimethyl-(1 theta)-bicyclo [3.1.1] hept-2-en. Alcohols were the primary volatile compounds, followed by lipids and ketones (acids, ketones, alkenes, and phenols were predominant). Alcohols, mainly from the oxidative degradation of polyunsaturated fatty acids, are the secondary lipid oxidation products [18]. Esters are obtained through the esterification of low-grade saturated fatty acids and saturated fatty alcohols. Most esters have fruity and floral flavors, which can reduce the pungency of fatty acids and the bitterness of amino acids [19]. Ketones are formed mainly via the oxidation of unsaturated fatty acids and the degradation of amino acids. Most ketones have a distinctive light and fruity flavor [20]. The harmonious coexistence of these volatile compounds enriches the characteristic aroma of fermented *D. officinale* juice.

### Untargeted metabolomics PCA analysis

**Fig. 3:**
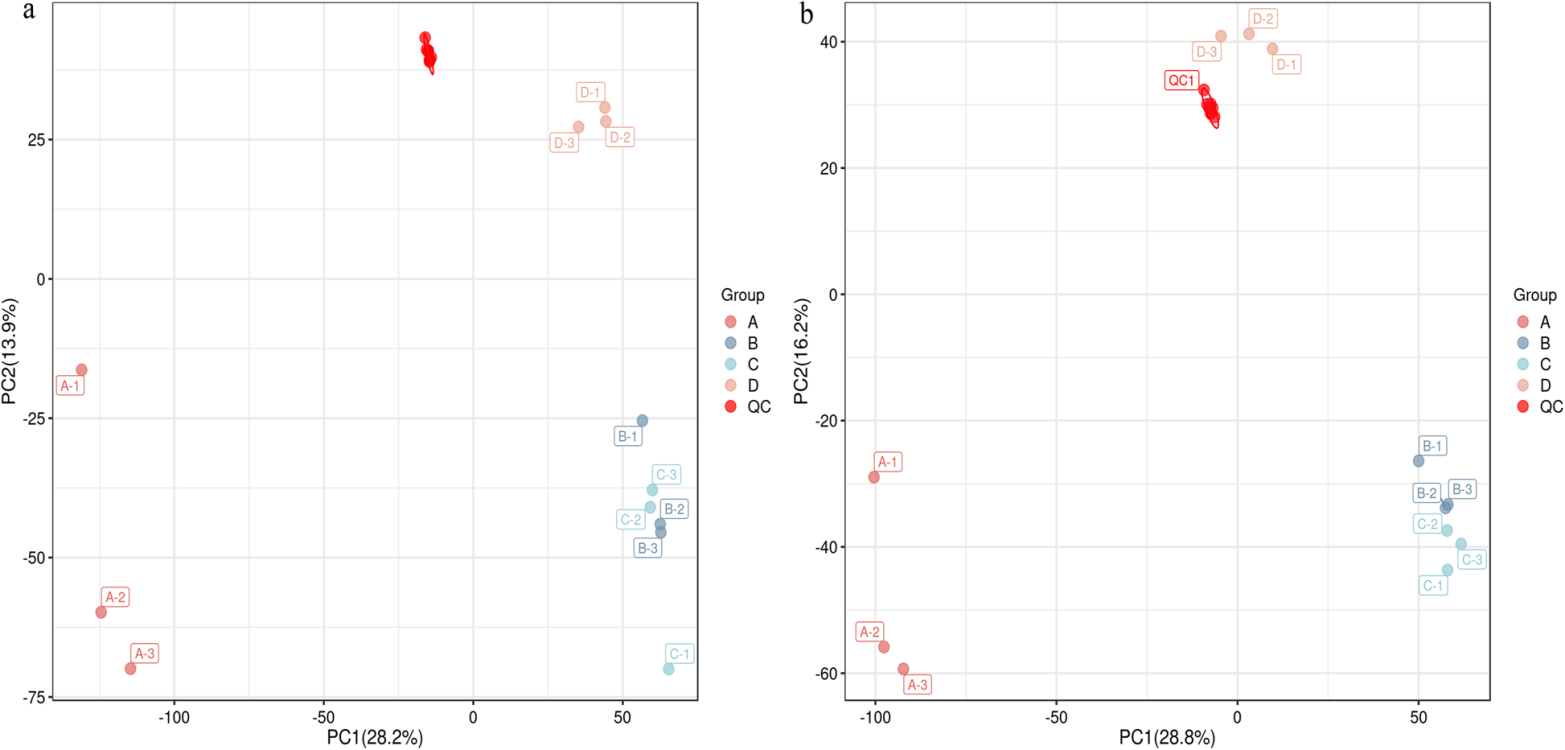
PCA score plots for QC samples in the positive ion mode (**a**) and negative ion mode (**b**)

**Fig. 4:**
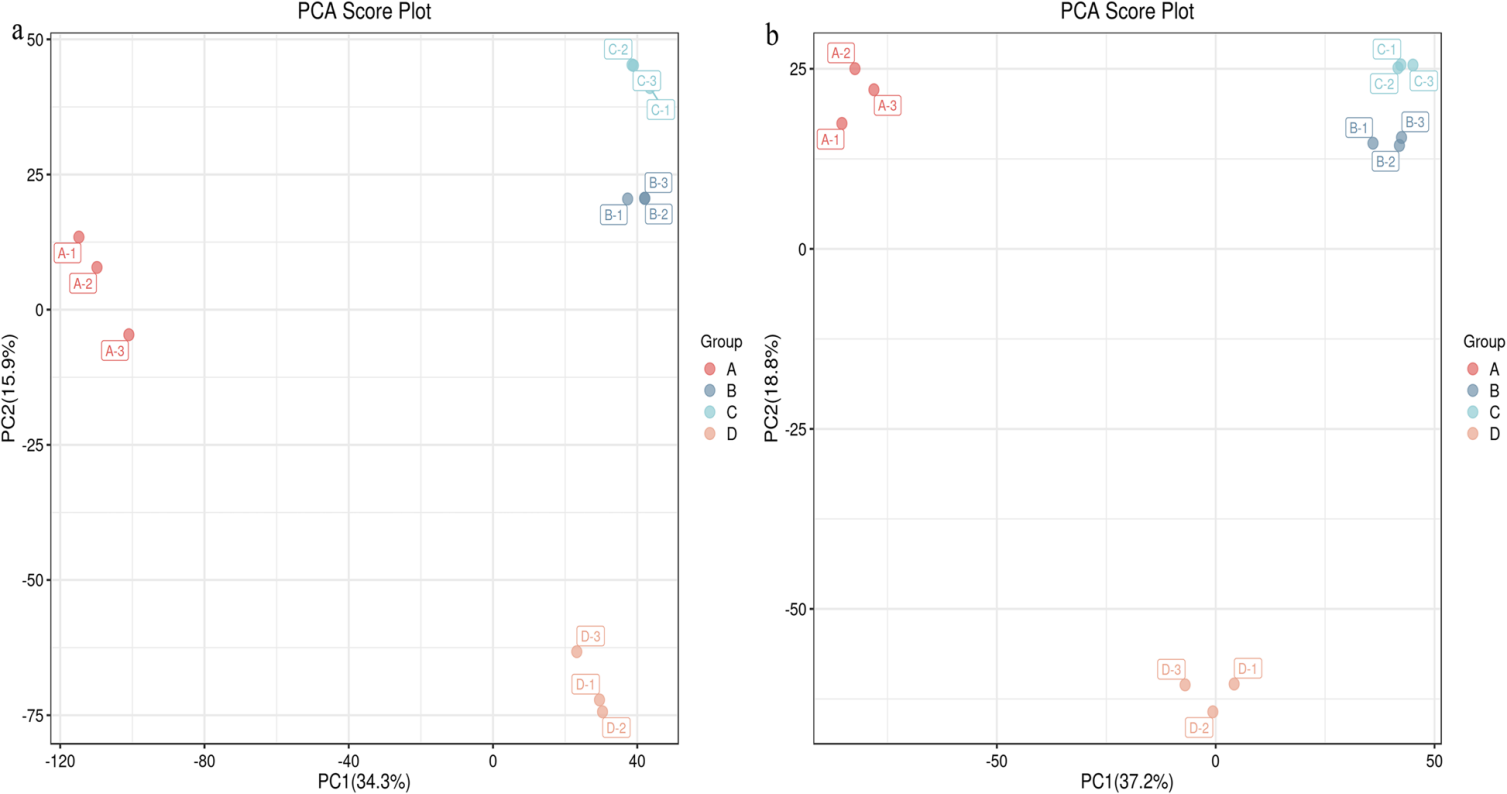
PCA scores of different D. officinale juice samples in the positive ion mode (**a**) and negative ion mode (**b**)

Quality control (QC) is essential to obtain reliable and high-quality metabonomic data in mass spectrometry-based metabonomic research [21]. The denser the distribution of QC samples on the PCA analysis plot, the more reliable the data. The PCA scores of QC in the positive and negative ion modes are shown in Fig. 3 a and b, respectively. The clustering of QC sample distribution indicates high method stability and good data quality, reproducibility, and reliable results. The PCA scores of different *D. officinale* juice samples in the positive and negative ion modes are shown in Fig. 4 a and b, respectively. The PCA modeling analysis was performed using uninoculated *D. officinale* juice A as the control group. The PCA results showed that the contribution rates of PC1 and PC2 in the positive ion mode were 34.3% and 15.9%, respectively (cumulative contribution rate; 50.2%). Similarly, the contribution rates of PC1 and PC2 in the negative ion mode were 37.2% and 18.8%, respectively (cumulative contribution rate; 56%). This result indicates that the separation trend between different inoculums of *D. officinale* juice was significant and could reflect the differences in metabolites between different samples [22].

### Untargeted metabolomics OPLS-DA analysis

**Fig. 5:**
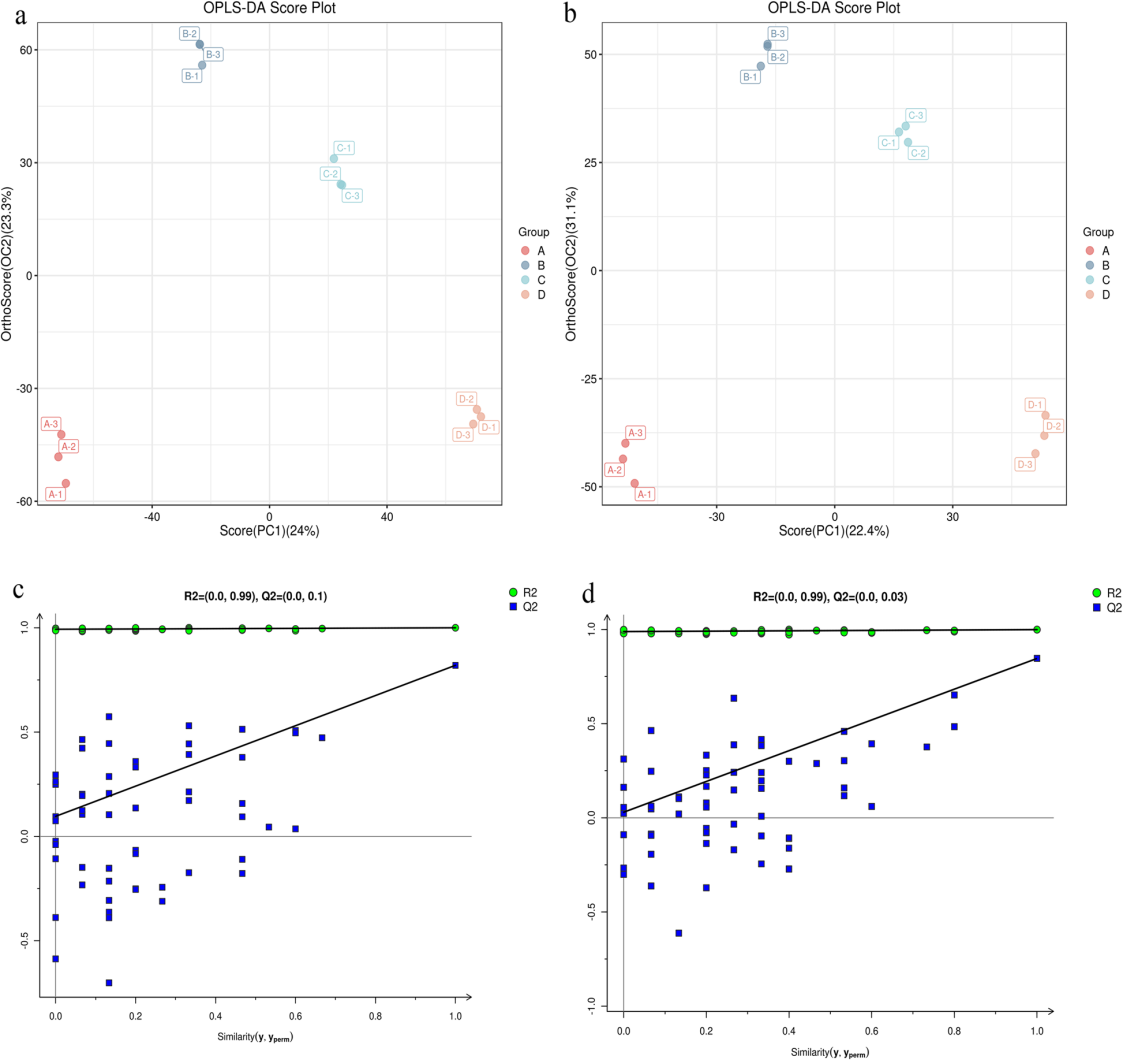
Plots of OPLS-DA scores for different groups of metabolites in the positive and negative ion modes **a**, **b**, and substitution tests **c**, **d**

In the PCA model, the differential variables were spread over more principal components due to the influence of relevant variables. This was unsuitable for finding the differences between the sample groups and screening for valid differential metabolites. The orthogonal projections to latent structures discriminant analysis (OPLS-DA) method can effectively reduce the complexity of the model and enhance the explanatory power of the model without reducing the predictive power of the model, thus maximizing the view of differences between groups [23]. The aggregation of each group of samples was better when uninoculated *D. officinale* juice in group A was used as the control group (R_X_^2^ = 0.579, R_Y_^2^ = 1.000 and Q^2^ = 0.820 in the positive ion mode and R_X_^2^ = 0.630, R_Y_^2^ = 0.999 and Q^2^ = 0.846 in the negative ion mode) (Fig. 5). The Q2 values of the model predictability were close to 1, indicating that the OPLS- DA model was more stable and reliable [24]. The position of all blue Q2 points in the OPLS-DA replacement test diagram was lower than those of the original blue Q2 points on the right, indicating the reliability and efficacy of the results.

### Differential metabolite screening

Group A of unfermented *D. officinale* juice was used as the control group, and the single *S. fibuligera* group B, single *L. paracasei* group C, and *S. fibuligera* + *L. paracasei* group D were used as the test groups. A total of 148 differential metabolites (76 up-regulated and 72 down-regulated) were screened in group B of *S. fibuligera* fermented *D. officinale* juice when the OPLS-DA first principal component variable importance value projection VIP > 1 and *P*-value < 0.05 (Table 1). Similarly, 155 differential metabolites (75 up-regulated and 80 down-regulated were screened in group C of *L. paracasei* fermented *D. officinale* juice. A total of 136 differential metabolites (75 up-regulated and 61 down-regulated) were screened in group D (*S. fibuligera* + *L. paracasei* fermentation*)*. The sample of *D. officinale* juice fermented with *L. paracasei* had the highest number of down-regulated differential metabolites, while the sample of *D. officinale* juice fermented with mixed culture had the lowest number of down-regulated differential metabolites. The transformation of these metabolites significantly changed the flavor of *D. officinale* juice [25].

**Table 1:** The Addition amount of fermentation strain

Inoculation volume (%)	A	B	C	D
*L. paracasei*	0	0	6	4
*S. fibuligera*	0	6	0	2

*Note*: *L*. is an abbreviation for Lactobacillus, and *S*. is an abbreviation for Saccharomycopsis

**Fig. 6:**
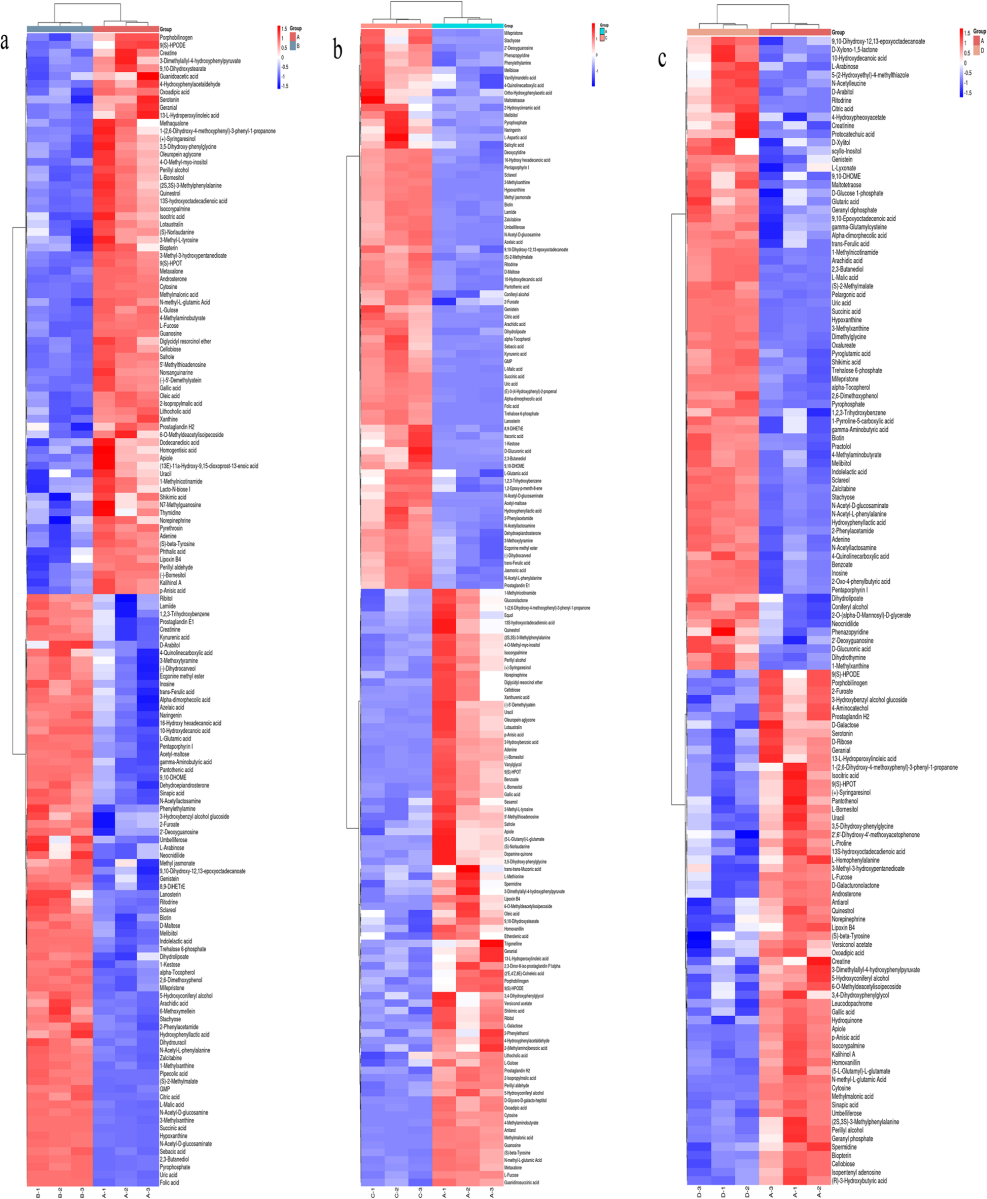
**a** shows the heat map of BvsA differential metabolites, **b** shows the heat map of CvsA differential metabolites, **c** shows the heat map of DvsA differential metabolites

In the thermographic cluster analysis (Fig. 6), the redder the color, the higher the expression amount and abundance, and the bluer the color, the lower the expression amount and abundance. The color significantly changed between the test group and the control group, and the expression of metabolites was significantly different. The differential metabolites screened from the fermentation group mainly included alcohols, organic acids, amino acids and peptides and their analogs, nucleic acids and their derivatives, carbohydrates, benzene compounds, terpenoids, alkanes, amines, ketones, and glycosides in 11 classes of compounds. Thirteen, 12, and 16 types of amino acids and peptides and their analogs are shown in Fig. 6a, b, and c, respectively. The following differential metabolites were present in groups a and b but not in group c: 5'-deoxy-5'-thionucleosides, flavones, glycosyl compounds, phenylacetaldehyde, porphyrins, purine ribonucleotides, pyrimidine 2'-deoxyribonucleosides, quinoline carboxylic acids, steroids and steroid derivatives, safrole, 3-methyl-L-tyrosine, budesonide methyl ester, (-)-5'-demethylation, (S)-norlaudanine, 9,10-dihydroxystearate, (-)-5'-demethylation, guanosine, Oleuropein aglycone, and Lamiide. Furthermore, the following differential metabolites were present in group a but not in groups b and c:2-benzopyrans, pyrethrin, and N7-methylguanosine. Also, 2-phenyl ethanol, trigonelline, L-methionine, and 1,2-Epoxy-p-menth-8-ene were present in group b but not in groups a and c. Finally, anilides, indolyl carboxylic acids, and derivatives, amino catechol, oxalureate, D-glucuronolactone, 2-Oxo-4-phenyl butyric acid, geranyl phosphate, geranyl diphosphate, isopentenyl adenosine, and D-galactose were present in group c but not in groups a and b. These results suggest that the co-culture of *L. paracasei* and *S. fibuligera* can inhibit the expression of some metabolites and enhance the composition of some metabolites. These changes in differential metabolites are essential for flavor formation in fermented beverages [12].

**Fig. 7:**
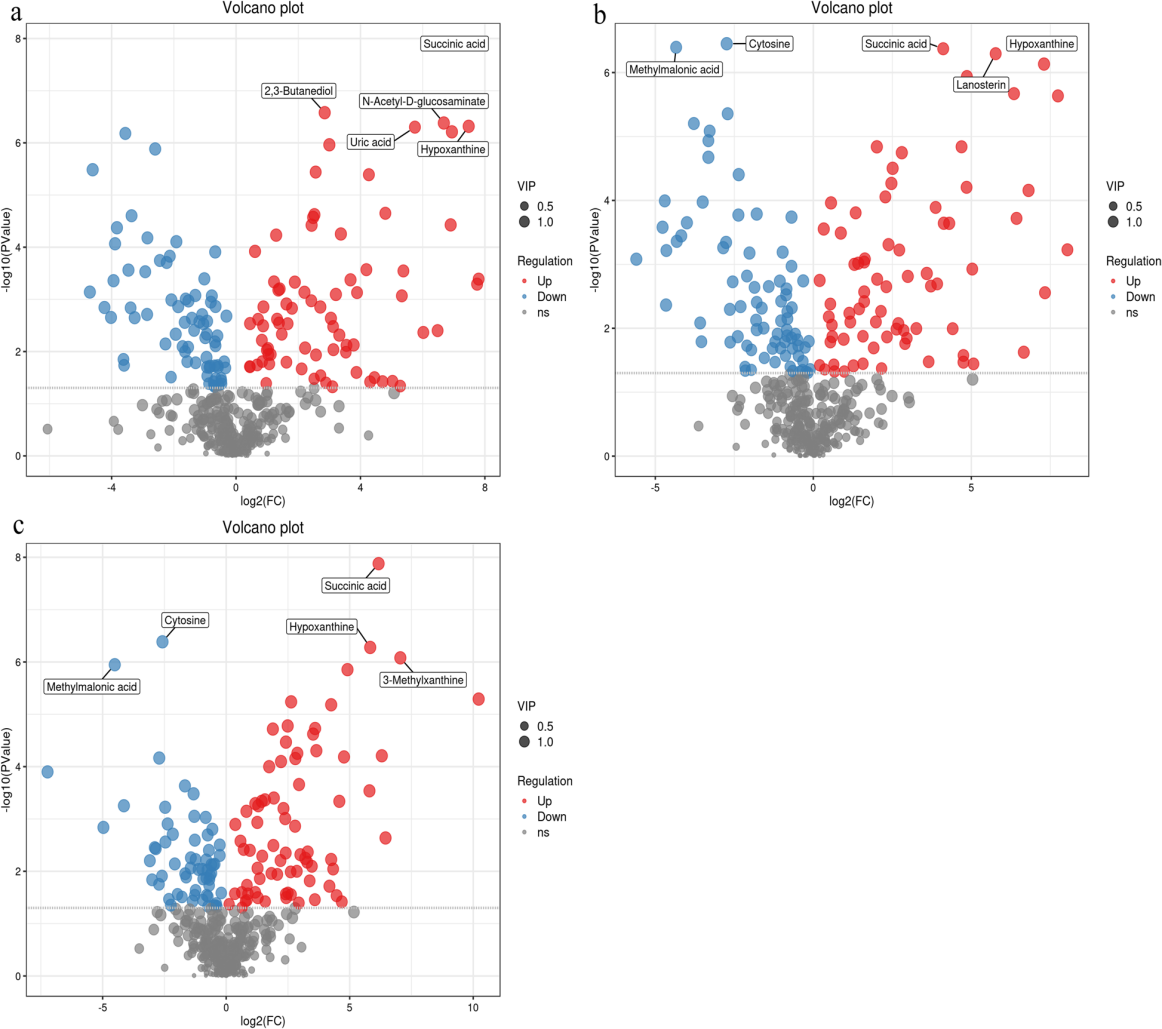
**a** is for the compound volcanoes of B vs. A, **b** is for the compound volcanoes of B vs. A, **c** is for the compound volcanoes of C vs. A

Each point in the volcano plot represents a metabolite, and the abscissa represents the logarithm of Log_2_, the quantitative difference multiple of a metabolite in two samples (Fig. 7). The ordinate represents the logarithm of—log_10_ of P value. The larger the absolute value of the horizontal coordinate, the greater the fold difference in the expression of a metabolite between two samples. The larger the vertical coordinate value, the more significant the differential expression and the more reliable the differentially expressed metabolites screened. The red and blue points in the graph represent up-regulated, and down-regulated differentially expressed metabolites, respectively. The grey points represent the metabolites detected without complying with the screening filtering parameters. The five most significantly up-regulated compounds were 2,3-butanediol, succinic acid, N-acetyl-D-glucosamine, uric acid, and hypoxanthine (Fig. 7a). The three most significantly up-regulated compounds were wool sterols, succinic acid, and hypoxanthine, and the two most significantly down-regulated compounds were cytosine and methylmalonic acid (Fig. 7b). The three most significantly up-regulated compounds included 3-methylxanthine, succinic acid, and hypoxanthine were the, and the two most significantly down-regulated compounds were cytosine and methylmalonic acid (Fig. 7c). Notably, 2,3-Butanediol is one of the most valuable liquid fuels and can be used as a flavor additive. The fuel has strong UV absorption and antioxidant properties. [26]. Succinic acid is a product and intermediate of anaerobic metabolism of many organisms and tricarboxylic acid cycle. It is used as an intermediate chemical in the production of cosmetics, pharmaceuticals, preservatives, herbicides, detergents, etc. [27]. N-Acetyl-D-Glucosamine (N-AcGA) is a highly sweet reducing monosaccharide used in the treatment of rheumatoid arthritis. Additionally, it is used as an antioxidant in foods, an additive in infants’ and children’s food, and a sweetener for people with diabetes N [28]. Uric acid, a scavenger of oxygen free radicals, helps maintain blood pressure stability, develops resistance to oxidative stress, and inhibits lipid peroxidation. Hypoxanthine is a natural purine base produced during purine catabolism and then converted into xanthine and uric acid. It can act as a precursor to uric acid and an intermediate in purine degradation by removing reactive oxygen species through the action of xanthine oxidase [29]. Lanosterol, a tetracyclic triterpenoid, is an essential intermediate in cholesterol biosynthesis and raw material for the synthesis of vitamin D and steroid-like hormones [30]. Cytosine is one of the primary bases for nucleic acid synthesis. The down-regulation of cytosine is associated with the synthesis of nucleic acids and their derivatives. Supplementation of food nucleic acids can significantly improve body immunity, anti-oxidation, influence fat metabolism, and maintain normal intestinal flora [31].

### Analysis of differential metabolite KEGG pathways

KEGG pathway analysis showed that 167 metabolic pathways were enriched in group B vs. A (40 were significantly enriched);170 metabolic pathways were enriched in group C vs. A (37 were significantly enriched and 147 metabolic pathways were enriched in group D vs. A (43 were significantly enriched) (*p* < 0.05). The results of the extremely r. The vertical and horizontal coordinates represent the metabolic pathways and the impact values enriched in different metabolic pathways. The higher the impact value, the higher the contribution of the metabolite detected in that pathway. The color represents the *P*-value; the redder the *P*-value, the less the *P*-value, and the bluer the *P*-value, the larger the *P*-value. The smaller the *P*-value, the more significant the impact of the detected differential metabolite in that pathway. Amino, organic, and nucleic acids were significantly up-regulated in the metabolite pathways, especially glutamic acid. The decomposition of amino acids significantly affects the flavor of fermentation broth [13]. Organic acids positively affect flavor presentation in sour drinks, with L-malic and citric acid being significantly up-regulated in several metabolic pathways. L-malic and citric acid have soft and intense acidity that enhances the product's mellowness [32].

**Fig. 8:**
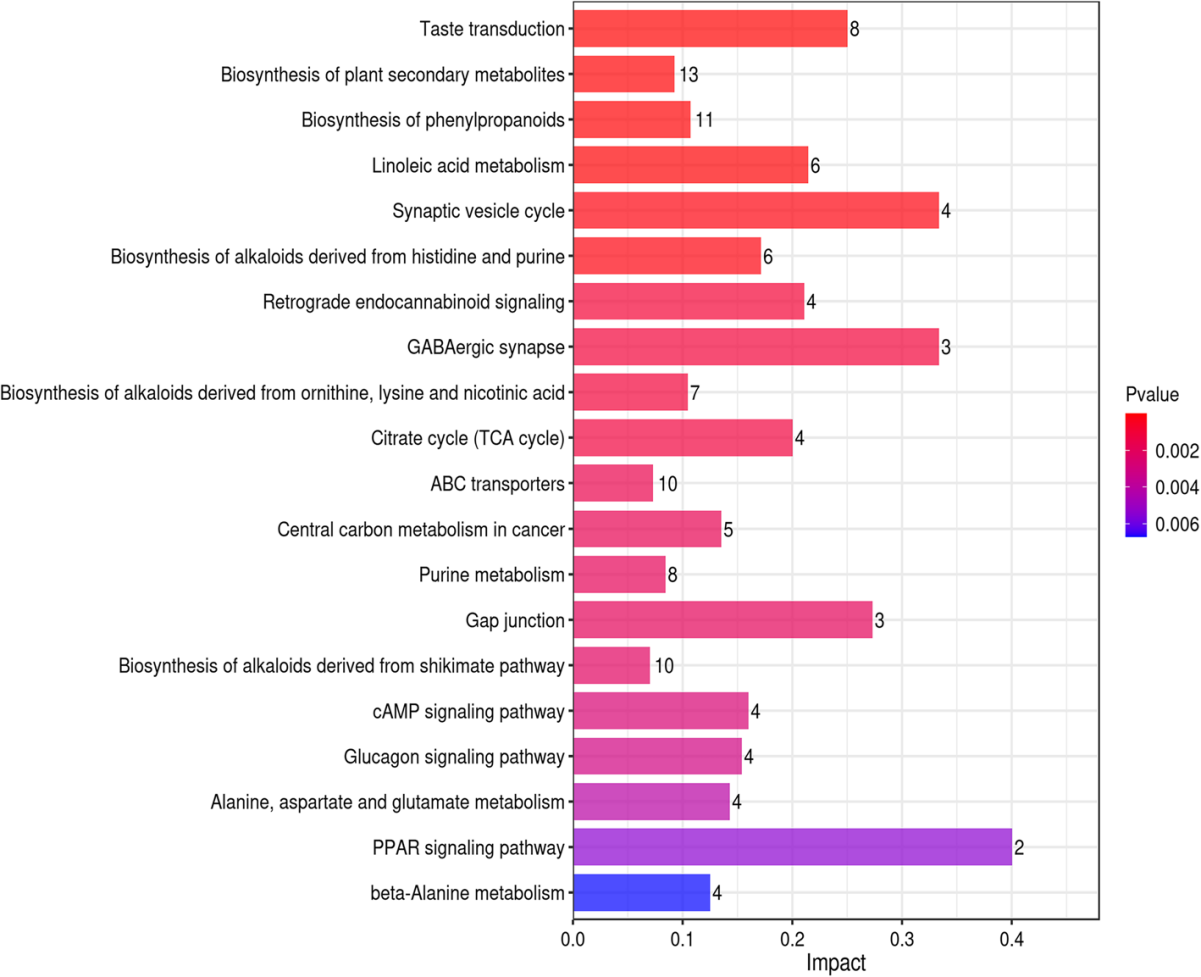
Histogram of factors influencing the B vs. A metabolic pathway

**Fig. 9:**
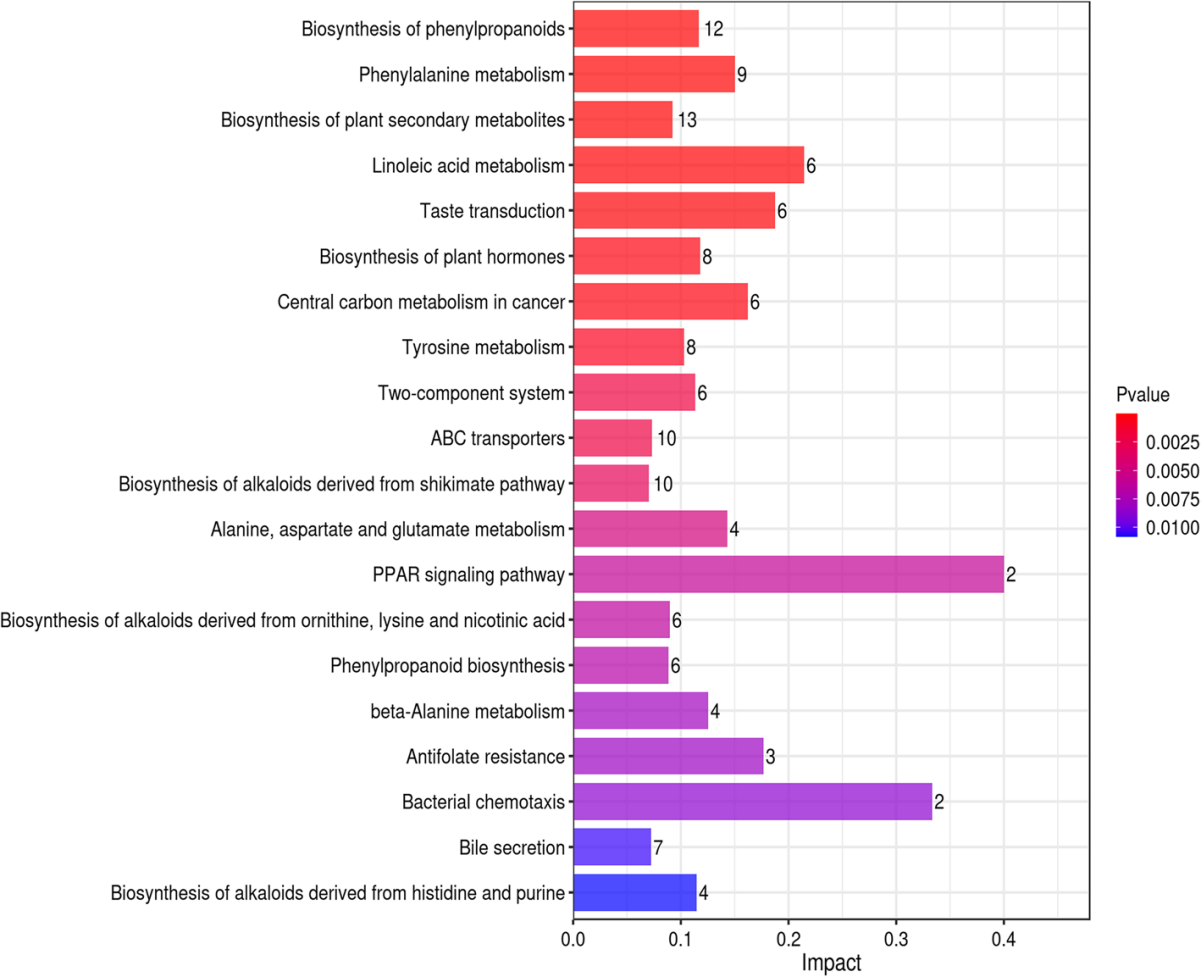
Histogram of factors influencing the C vs. A metabolic pathway

**Fig. 10:**
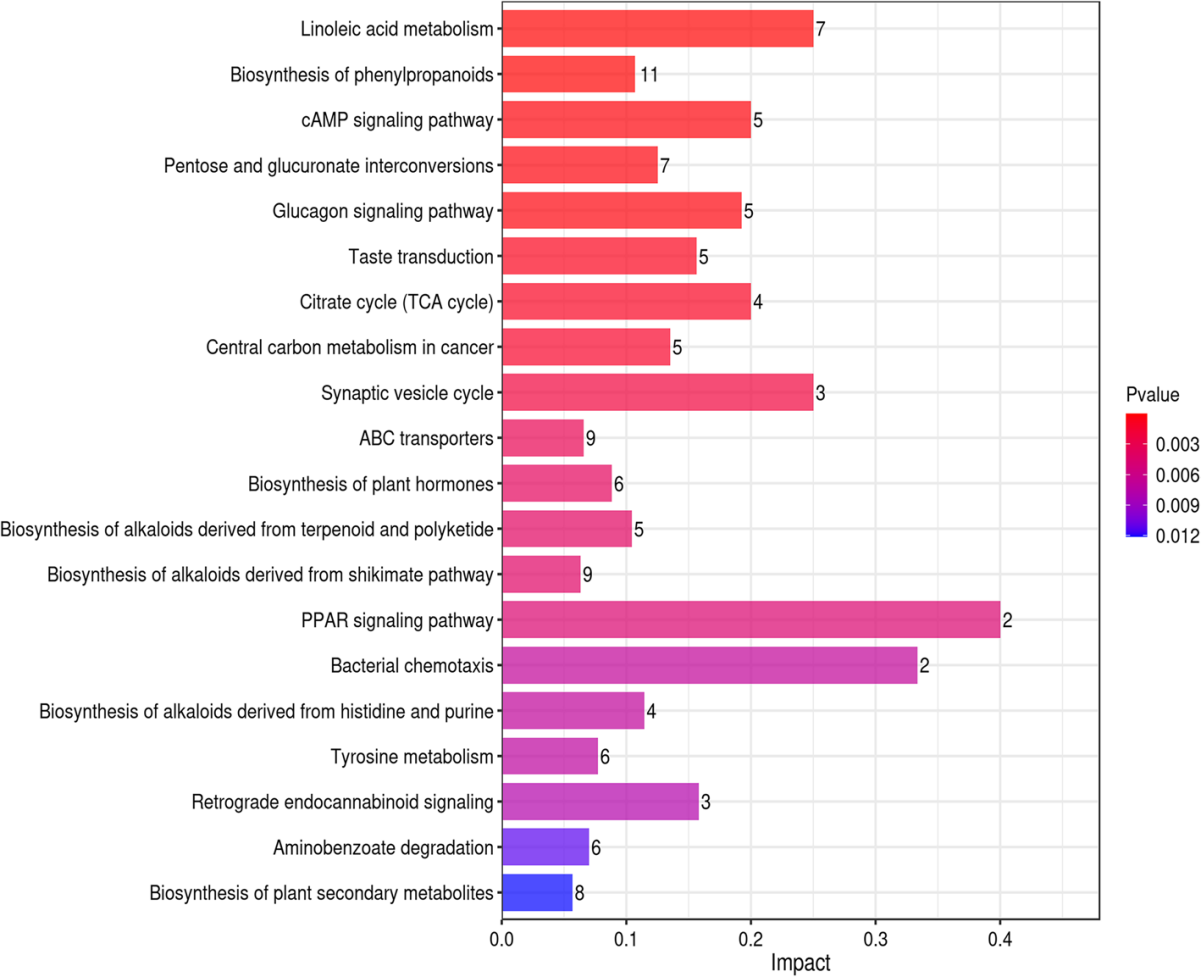
Histogram of factors influencing the D vs. A metabolic pathway

The differential metabolites of *D. officinale* juice fermented with single *S. fibuligera* were significantly different from those in the *D. officinale* juice fermented with single *L. paracasei* in 7 of the 20 most significant metabolic pathways (Fig. 8, 9, and 10). There were five differences between the *D. officinale* juice fermented with single *S. fibuligera* and the *D. officinale* juice fermented with *S. fibuligera* + *L. paracasei*, while there were seven differences between the *D. officinale* juice fermented with single *L. paracasei* and the *D. officinale* juice fermented with *S. fibuligera* + *L. paracasei*. However, three pathways (pentose and glucuronate interconversions, biosynthesis of alkaloids derived from terpenoid and polyketide, and aminobenzoate degradation)in the *D. officinale* juice fermented with the mixed culture of *S. fibuligera* + *L. paracasei* were not found in the most significant 20 pathways in the *D. officinale* juice fermented with single *S. fibuligera* and the *D. officinale* juice fermented with single *L. paracasei*. The interconversion of pentose and glucuronic is a metabolic process of carbohydrates and compounds. Terpenoids are primary active substances with several physiological activities, such as anti-inflammatory, anticancer, anti-bacterial, and anti-viral [33]. Polyketones are mainly used for synthesizing anticancer drugs and lipid-lowering drugs. The degradation of aminobenzoic acid facilitates the synthesis of catechol and 3-methyl catechol.

### Analysis of secondary metabolite synthesis

The addition of strains to ferment *D. officinale* juice facilitated the synthesis of secondary metabolites. Seven organic acids, four fatty acids, three carbohydrates, three amino acids, three benzene compounds, two imidazoles, two vitamins, two terpenoids, two flavonoids, one alkaloid, one ester, and one other metabolite were significantly up-regulated after fermentation (Table 2). Among the up-regulated metabolites, succinic acid, citric acid, and trans-ferulic acid were found in all three sample groups, while 2-hydroxycinnamic acid and salicylic acid were specific to the single *L. paracasei* fermentation group C. Protocatechuic acid and Shikimic acid were significantly up-regulated in the *S. fibuligera* + *L. paracasei* fermentation group D. The log_2_FC value of L-malic acid was highest in the mixed fermentation group. The down-regulated metabolites, such as methyl jasmonate, jasmonic acid, and sinapic acid, in the mixed fermentation group utilized D-maltose and promoted the synthesis of D-glucose-1-phosphate, but no amino acid compound was up-regulated.

**Table 2:** Up-regulation and down-regulation of differential metabolites in the test group

Comparison	Up	Down	Total
B vs. A	76	72	148
C vs. A	75	80	155
D vs. A	75	61	136

### Analysis of key metabolite pathways in the mixed culture groups

**Fig. 11:**
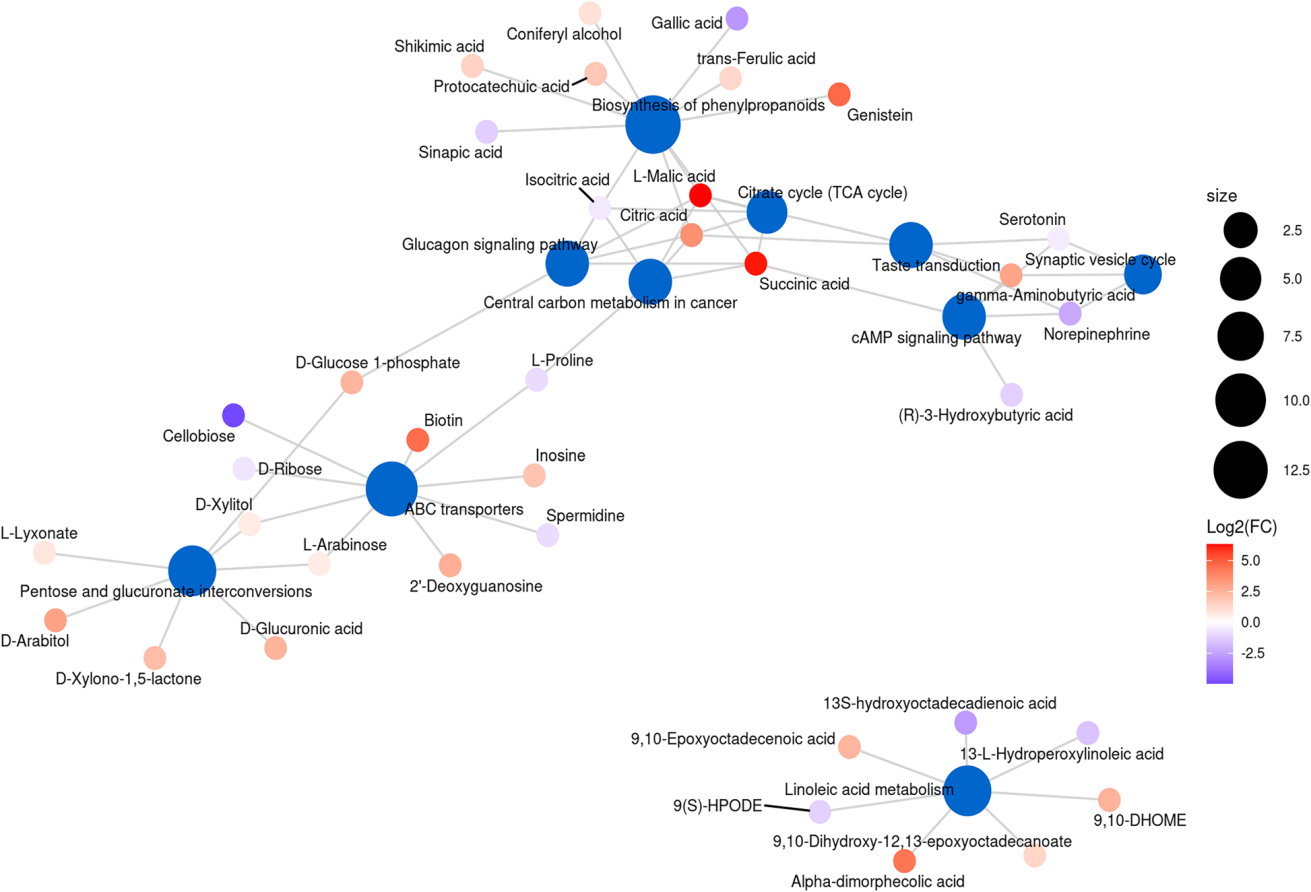
Differential metabolite network diagram

The metabolic pathways of the differential metabolites in the fermented *D. officinale* juice from *S. fibuligera* + *L. paracasei* were analyzed. The blue and other dots in Fig. 11 represent pathway and metabolites, respectively. The size of the pathway dot represents the number of metabolites connected to it. The metabolite dot shows the size of log2FC value through the gradient color. Ten key metabolic pathways, including linoleic acid metabolism, phenylpropanoid biosynthesis, cAMP signaling pathway, pentose and glucuronate interconversions, glucagon signaling pathway, taste transduction, citrate cycle (TCA cycle), central carbon metabolism in cancer, synaptic vesicle cycle, and ABC transporters were obtained. Twenty-three metabolites were significantly up-regulated in the ten critical metabolic pathways (9,10-DHOME, D-Xylono-1,5-lactone, gamma-minobutyric acid, genistein, succinic acid, trans-ferulic acid, L-malic acid, citric acid, coniferyl alcohol, protocatechuic acid, shikimic acid, D-glucose 1-phosphate, biotin, inosine, D-xylitol, L-arabinose, 9,10-dihydroxy-12,13-epoxyoctadecanoate, 9,10-epoxyoctadecenoic acid, D-lyxonate, 2'-deoxyguanosine, D-arabinol, and D-gulucronic acid) (Fig. 11, 23). However, 11 acid metabolites were significantly up-regulated in the metabolic pathways, thus enhancing *D. officinale* juice flavor formation.

## Discussion

The results showed that the addition of *S. fibuligera* group B and the fermentation of *D. officinale* juice in co-culture group D with *L. paracasei* and *S. fibuligera* significantly increased the content of alcohols, including isoamyl alcohol, linalool, phenylethanol and isobutanol. Furthermore, the content of ethyl L(-)-lactate was higher in the *L.paracasei* + *S. fibuligera* fermentation group D than in *L. paracasei* monobacterial fermentation group C and *S. fibuligera* monobacterial fermentation group B, indicating that the co-culture of the *L. paracasei* + *S. fibuligera* increases the content of ethyl L(-)-lactate in the *D. officinale* juice. The esterification reaction between acids and alcohols catalyzed by esterases increased the content of ethyl L(-)-lactate. The main metabolites of *S. fibuligera* are alcohols. *L. paracasei* is mainly responsible for acidifying *D. officinale* juice [4, 34].

Untargeted metabolomics based on liquid chromatography-mass spectrometry combined with multivariate statistical analysis methods has been used in recent years to screen differential metabolites and analyze microbial metabolites. Zhang [35] screened and identified potential antioxidant and anti-inflammatory components using UPLC-Q-TOF–MS-based untargeted metabolomics and correlation analysis. That was the first study to systematically and effectively track antioxidant and anti-inflammatory components in fermented bee pollen. Also, Long [36] showed that pu-erh tea undergoes a stack fermentation process using a comprehensive chemical analysis combined with untargeted and targeted metabolomics. In this study, several biologically active compounds, such as organic acids, fatty acids, amino acids, alkaloids, flavonoids, vitamins, terpenoids, L-malic acid and succinic acid were detected in fermented *D. officinale* juice via untargeted metabolomics approach. These compounds are key intermediates of the tricarboxylic acid cycle and have anti-bacterial, anti-inflammatory, and antioxidant effects. Organic acids can also reduce unpleasant odors in raw materials. Besides, the bioactive substances have antioxidant, anti-bacterial, and cytotoxic activities. Alkaloids can reduce obesity by interacting with adipose stem cells and preadipocytes. Terpenoids have significant cytotoxic activity, while phenolic acids can improve cardiovascular diseases, including hypertension [37, 38].

## Conclusions

In this study, the headspace solid-phase microextraction-gas combination technology was used to analyze the changes in the volatile components before and after fermentation of *D. officinale* juice. The differential metabolites were screened using the untargeted metabolomics of liquid chromatography-mass spectrometry combined with multiple statistics through analytical methods. The alcohol contents were significantly increased after fermentation. The co-culture increased ethyl L(-)-lactate content. Additionally, *D. officinale* juice fermented with *L. paracasei* + *S. fibuligera* prevented the downregulation of some metabolites. *D. officinale* juice fermented with *L. paracasei* + *S. fibuligera* also enhanced Pentose and glucuronate interconversions and biosynthesis of alkaloid. The three metabolic pathways derived from terpenoid and polyketide and Aminobenzoate degradation are involved in many biological activities, such as synthesis of terpenes, polyketones and phenols. Synthesis of substances, rich in the flavor composition of *D. officinale* juice. Therefore, this study provides a theoretical basis for flavor formation analysis in *D. officinale* and analysis of functions of metabolites in fermented *D. officinale*.

## Materials and methods

### Materials and reagents

*Dendrobium officinale* was obtained from Guizhou Qiansheng Taijiang Poverty Alleviation Industry Development Co., LTD (Guizhou, China). *Saccharomycopsis fibuligera* FBKL2.8DCJS1, was isolated and screened from Wild Grape (Guizhou, China), and conserved in China Center for Type Culture Collection (Wuhan, China), No. CCTCC NO: M2020891. *Lactobacillus paracasei* FBKL1.3028, was isolated and screened from White Sour Soup (Guizhou Province, China), and conserved in China Center for Type Culture Collection (Wuhan, China), No. CCTCC NO: M2020156. MRS broth medium, MRS agar medium, YEPD medium, and YPD medium were obtained from Shanghai Bo Microbiology Technology Co., Ltd. (Shanghai, China). Liquid malt (100% malt) was obtained from Gansu Hongli Biotechnology Co., Ltd. (Gansu, China). Glucose was obtained from Hebei Jinfeng Starch Glycol Co., Ltd. (Hebei, China). α- High temperature amylase and saccharifying enzyme were obtained from Solarbio (Beijing, China). Pectinase was obtained from Manson Trading Co., Ltd. (France); 2-octanol, German Dr Ehrenstorfer GmbH (Germany). Acetonitrile Thermo; Formic acid was obtained from TCI. Ammonium formate was obtained from Sigma-Aldrich (Shanghai, China).

### Fermentation of D. officinale juice

*S. fibuligera* in glycerol tubes at -80 °C was activated in YPD medium at 28 °C for 48 h, while *L. paracasei* was activated in MRS solid medium at 37 °C for 48 h. The activated strains were inoculated into sterilized and cooled liquid malt extract solution (128 g of liquid malt extract, 875 mL of distilled water in four 500 mL conical flasks at 115 °C for 20 min). *S. fibuligera* was fixed at 28 ℃ for 36 h at 160 r/min while *L. paracasei* was fixed at 37 ℃ for 36 h at 160 r/min. *D. officinale* fresh strips were dried and crushed through a 40-mesh sieve, then 60 g of *D. officinale* powder was added to 4 L of distilled water and boiled for 1 h. α-high temperature amylase (4 g) was added to the pot and enzymatically digested at 95 ℃ for 1 h. Pectinase (2 g) and 2 g of glycosylase were also added to the pool and enzymatically digested at 55 ℃ for 1 h. Glucose (300 g) was then added to the sample, dissolved, filtered, encapsulated in 12 bottles, and sterilized at 115 ℃ for 20 min (unfermented *D. officinale* juice). The juice was cooled, then inoculated with 6% *L. paracasei*, 6% *S. fibuligera*, 4% *L. paracasei*, and 2% *S. fibuligera* (three replicates for each inoculation) (Table 3). The unfermented *D. officinale* juice was used as a blank control. After inoculation, the juice was incubated at 37 ℃ for 24 h, fermented at 28 ℃ for 15 days, and post-ripened at 4 ℃ for 30 days to obtain the sample.

**Table 3:** Analysis of secondary metabolite synthesis in fermented *D. officinale* juice

Classify	Name	m/z	Rt/s	formula	log_2_FC		
					B vs. A	C vs. A	D vs. A
organic acids	Succinic acid	116.9854	144.7	C_4_H_6_O_4_	6.19	4.11	6.17
	Citric acid	191.0198	153	C_6_H_8_O_7_	6.02	6.35	3.59
	trans-Ferulic acid	193.0505	335.5	C_10_H_10_O_4_	1.12	1.61	1.28
	2-Hydroxycinnamic acid	165.0552	504.3	C_9_H_8_O_3_	-	1.98	-
	Salicylic acid	137.0238	400.9	C_7_H_6_O_3_	-	0.2	-
	Protocatechuic acid	153.0192	828.4	C_7_H_6_O_4_	-	-	1.83
	Shikimic acid	173.0454	104.9	C_7_H_10_O_5_	-	-	1.44
fatty acids	*L*-Malic acid	134.0177	77.5	C_4_H_6_O_5_	4.27	2.28	6.3
	Methyl jasmonate	207.1373	634.9	C_13_H_20_O_3_	2.89	6.66	-
	Sinapic acid	207.0648	511.9	C_11_H_12_O_5_	0.88	-	-
	Jasmonic acid	193.1219	706.7	C_12_H_18_O_3_	-	2.33	-
carbohydrates	Trehalose 6-phosphate	421.0747	78.5	C_12_H_23_O_14_P	6.48	7.33	5.8
	*D*-Maltose	342.1391	141.9	C_12_H_22_O_11_	2.2	2.38	-
	*D*-Glucose 1-phosphate	261.0361	90	C_6_H_13_O_9_P	-	-	2.41
amino acids	Pipecolic acid	130.0854	142.6	C_6_H_11_NO_2_	2.71	-	-
	*L*-Glutamic acid	130.0486	167.9	C_5_H_9_NO_4_	2.22	0.67	-
	*L*-Aspartic acid	132.0304	81.5	C_4_H_7_NO_4_	-	2.86	-
benzene compounds	5-Hydroxyconiferyl alcohol	179.0709	286.6	C_10_H_12_O_4_	1.29	-	-
	(*E*)-3-(4-Hydroxyphenyl)-2-propenal	131.0488	650.8	C_9_H_8_O_2_	-	3.59	-
	Coniferyl alcohol	181.0862	457.3	C_10_H_12_O_3_	-	0.6	0.96
imidazoles	3-Methylxanthine	166.0496	336.9	C_6_H_6_N_4_O_2_	6.94	7.3	7.05
	1-Methylxanthine	166.0497	353.3	C_6_H_6_N_4_O_2_	2.43	-	2.39
vitamins	alpha-Tocopherol	430.2402	495.7	C_29_H_50_O_2_	6.9	6.81	6.45
	Pantothenic acid	218.1036	195.8	C_9_H_17_NO_5_	2.57	2.63	-
terpenoids	Lanosterin	425.2556	814.5	C_30_H_50_O	5.28	5.77	-
	Sclareol	291.2669	790.6	C_20_H_36_O_2_	1.89	2.01	1.74
flavonoids	Genistein	271.2257	686	C_15_H_10_O_5_	4.71	4.41	4.68
	Naringenin	271.061	647.1	C_15_H_12_O_5_	1.66	2.14	-
alkaloid	Ecgonine methyl ester	182.1171	693.7	C_10_H_17_NO_3_	1.03	1.14	-
ester	Benzoate	121.0294	524.3	C_7_H_6_O_2_	-	-	2.95
other	Geranyl diphosphate	314.0903	250.4	C_10_H_20_O_7_P_2_	-	-	1.91

### HS–SPME–GC–MS analysis

Each sample of the fermented and unfermented *D. officinale* juice was treated in triplicate. The volatile components of the *D. officinale* juice were determined via headspace solid-phase microextraction and gas chromatography using 7890A/5975C gas chromatography-mass spectrometer equipped with PAL automatic sampler and DB-WAX (30 m × 0.25 mm; i.d.,0.25 μm) capillary chromatographic column. Briefly, 8 mL of the sample, 2 g of NaCl, and 1 μL of internal standard (2-octanol, 0.822 mg/mL by mass) were added to a 20 mL sample bottle, then the bottle was quickly sealed by tightening the cap. The sample was equilibrated at 45 °C for 10 min at 160 r/min until the gas–liquid aromatic substances in the bottle reached the equilibrium state. The extraction head was then inserted into the headspace of the sample bottle, and stirred for 45 min to achieve gas–solid and gas–liquid balance of the aromatic substances in the sample bottle. The extraction head was then inserted into the GC–MS injection port and thermally desorbed at 250 °C for 10 min, and the sample was injected in non-split mode. The column flow rate was 0.8 mL/min in a constant flow mode. The programmed temperature was increased from 40 °C to 120 °C at 10 °C/min and held for 3 min, then increased to 230 C at 20 C/min for 67 min. The ion source temperature, ion energy source, and mass scan range were 230 °C, 70 eV, and 20–350 u, respectively (ionization mode; electron ionization (EI)) [39].

### LC–MS analysis

First, 6 mL of supernatant from samples before and after fermentation were divided into three vials (2 mL each), then frozen in liquid nitrogen for further use. The samples were thawed at 4 ℃, vortexed for 1 min, and mixed evenly. An appropriate amount of sample was transferred into a 2 mL centrifuge tube, then 500 µL of methanol solution (stored at -20 ℃) was added to the sample and vortexed for 1 min. The sample was centrifuged at 12,000 rpm and 4 ℃ for 10 min, and the supernatant was transferred to a new 2 mL centrifuge tube. The supernatant was concentrated and dried, then 150 µL of 2-Amino-3-(2-chloro-phenyl)-propionic acid (4 ppm) solution prepared with 80% methanol–water (stored at -20 ℃) was added to redissolve the sample. The supernatant was filtered using a 0.22 µm membrane and transferred into the detection bottle for LC–MS detection.

Liquid chromatography conditions: The LC analysis was performed on a Vanquish UHPLC System (Thermo Fisher Scientific, USA) using an ACQUITY UPLC ® HSS T3 (150 × 2.1 mm, 1.8 µm) (Waters, Milford, MA, USA). The column was maintained at 40 ℃. The flow rate and injection volume were set at 0.25 mL/min and 2 μL, respectively. For LC-ESI ( +)-MS analysis, the mobile phases consisted of (B2) 0.1% formic acid in acetonitrile (v/v) and (A2) 0.1% formic acid in water (v/v). Separation was conducted under the following gradients: 0 ~ 1 min, 2% B2; 1 ~ 9 min, 2% ~ 50% B2; 9 ~ 12 min, 50% ~ 98% B2; 12 ~ 13.5 min, 98% B2; 13.5 ~ 14 min, 98% ~ 2% B2; 14 ~ 20 min, 2% B2. For LC-ESI (-)-MS analysis, the mobile phase consisted of acetonitrile (B3) and 5 mM of ammonium formate (A3). Separation was conducted under the following gradients: 0 ~ 1 min, 2% B3; 1 ~ 9 min, 2% ~ 50% B3; 9 ~ 12 min, 50% ~ 98% B3; 12 ~ 13.5 min, 98% B3; 13.5 ~ 14 min, 98% ~ 2% B3; 14 ~ 17 min, 2% B3 [40].

Mass spectrum conditions: Mass spectrometric detection of metabolites was performed on Q Exactive Focus (Thermo Fisher Scientific, USA) with an ESI ion source and simultaneous MS1 and MS/MS (Full MS-ddMS2 mode, data-dependent MS/MS) acquisition. The parameters were as follows: sheath gas pressure, 30 arb; aux gas flow, 10 arb; spray voltage, 3.50 kV and -2.50 kV for ESI ( +) and ESI (-), respectively; capillary temperature, 325 ℃; MS1 range, m/z 100–1000; MS1 resolving power, 70,000 FWHM; the number of data-dependent scans per cycle, 3; MS/MS resolving power, 17,500 FWHM; normalized collision energy, 30 eV; dynamic exclusion time, automatic [41].

### Volatile component data analysis

All tests were performed in triplicate. The experimental data were collated and graphed using Excel 2010. IBM SPSS Statistics, 26, and Origin 2021 were used for graphical plotting. The significance analysis was performed using the Duncan test. *P* < 0.05 was considered statistically significant. TBtools was used for plotting the heat maps.

### Differential metabolite analysis

The principal component analysis (PCA), partial least squares discriminant analysis (PLS-DA), and orthogonal partial least squares discriminant analysis (OPLS-DA) were performed using the R package Ropls [42] to reduce the dimensionality of the sample data. The score, loadings, and S-plot plots were used to demonstrate the differences in metabolite composition among the samples. The model was tested for overfitting using a permutation test. The functional pathways of the screened differential metabolites were enriched and topologically analyzed using MetaboAnalyst [43] software package. The enriched pathways were analyzed using the KEGG Mapper visualization tool for differential metabolite and pathway maps.

## Data Availability

All data generated or analyzed during this study have been included in this published article and its supplementary information files.

## Abbreviations

*D. **officinale*: *Dendrobium* *officinale*
*L. **paracasei*: *Lactobacillus* *paracasei*
*S. **fibuligera*: *Saccharomycopsis* *fibuligera*
